# *Bovine Viral Diarrhea* in Kazakhstan

**DOI:** 10.3390/v17101341

**Published:** 2025-10-05

**Authors:** Elvira Bashenova, Raikhan Nissanova, Vladimir Kirpichenko, Perizat Akshalova, Angelina Malysheva, Fariza Ikramkulova, Alena Cherusheva, Yergali Abduraimov, Aralbek Rsaliyev, Kunsulu Zakarya, Aisha Zharmukhametova, Saltanat Kuatbekova, Artyom Kuligin, Zhandos Abay, Zhibek Zhetpisbay, Seidigapbar Mamadaliyev, Ainur Nurpeisova, Markhabat Kassenov

**Affiliations:** 1 Kazakh Scientific Research Veterinary Institute LLP, National Holding QazBioPharm, 223 Rayymbek Avenue, Almaty 050016, Kazakhstan; elvirabashenova17@gmail.com (E.B.); vladimir.kirpichenko1992@gmail.com (V.K.); peri.akshalova@gmail.com (P.A.); fariza.ikramkulovaa@gmail.com (F.I.); zh.aisha.zh98@gmail.com (A.Z.); abai.zhandos15@gmail.com (Z.A.); nurai1005@gmail.com (A.N.); kasenovmarhabat@gmail.com (M.K.); 2 Almaty Branch of the National Center for Biotechnology, National Holding QazBioPharm, 14 Zhahanger Str., Almaty 050054, Kazakhstan; malysheva.angelina.alx@gmail.com (A.M.); neupokoyeva.as@gmail.com (A.C.); kuatbek9205@gmail.com (S.K.); kuligin.artyoom@gmail.com (A.K.); sdgap.mam@gmail.com (S.M.); 3 JSC National Holding QazBioPharm, 13/5 Korgalzhinskoe Highway, Astana 010000, Kazakhstan; e.abduraimov@qbp-holding.kz (Y.A.); a.rsaliyev@qbp-holding.kz (A.R.); krzakarya@gmail.com (K.Z.); 4 Department of Computer Science, Al-Farabi Kazakh National University, Al-Farabi Avenue 71, Almaty 050040, Kazakhstan; zhibekzhetpisbay@gmail.com

**Keywords:** *Bovine Viral Diarrhea*, diagnosis, reference serum, Kazakhstan, genotyping

## Abstract

*Bovine Viral Diarrhea Virus* (BVDV) is a globally important cattle pathogen causing substantial economic losses. In Kazakhstan, BVDV’s epidemiological status remains poorly characterized due to the absence of systematic surveillance. We carried out a cross-sectional study of cattle herds across Kazakhstan, using ELISA to detect anti-BVDV antibodies and RT-PCR to identify active infections. Positive samples underwent sequencing for phylogenetic analysis of circulating strains. Additionally, a standard reference serum panel was developed to measure virus neutralization titers (ND_50_) and to evaluate cross-neutralization with *Border Disease virus* (BDV). Antibodies against BVDV were prevalent, with seropositivity ranging from 28.89% to 96.13% across surveyed regions. Active BVDV infection was confirmed by RT-PCR in 17 animals. Phylogenetic analysis with 2 samples from Mangystau region classified the virus as BVDV2 genotype. The reference serum panel exhibited high neutralizing titers ND_50_ up to 1:286 against the local BVDV-1 isolate. Notably, these sera also neutralized BDV, albeit at lower titers ND_50_ 1:45. These findings provide crucial baseline epidemiological data and enhanced diagnostic tools for BVDV in Kazakhstan. They highlight the need for improved surveillance and will inform strategic control measures against this economically significant cattle disease.

## 1. Introduction

*Bovine Viral Diarrhea Virus* (BVDV) is a globally prevalent *Pestivirus* (family Flaviviridae) and remains one of the most consequential pathogens of cattle [1]. It causes a wide spectrum of clinical outcomes—from immunosuppression and reproductive failure to persistent infection and the fatal mucosal disease—making it a major threat to herd health [2,3,4]. The economic impact of BVDV is enormous: industry losses are estimated to exceed one billion dollars annually worldwide due to high calf mortality, increased treatment costs, and production losses [5,6,7]. By compromising animal welfare and productivity, BVDV imposes a heavy burden on livestock systems across the globe. Intensive control programs in many countries have achieved measurable success in reducing BVDV prevalence through strict biosecurity measures, systematic testing, removal of persistently infected (PI) animals, and vaccination [8,9,10]. Several European nations have even reached near-eradication of BVDV by adopting rigorous test-and-cull strategies [11,12,13]. In contrast, BVDV continues to circulate largely unchecked in regions with limited surveillance capacity. Central Asia, and the Republic of Kazakhstan in particular, remains a blind spot in the global BVDV landscape: despite Kazakhstan’s large cattle population and its strategic position bridging Europe and Asia, there is currently no national program for monitoring BVDV prevalence, genotypes, or diagnostic capacity. This gap leaves significant uncertainty about the true epidemiological status of pestiviruses in the region. Moreover, the unchecked circulation of BVDV—and its diagnostic interplay with the closely related BDV of small ruminants—poses challenges beyond just animal health [11]. Co-circulation of pestiviruses in sheep and goats (e.g., BDV) can confound diagnostics and even spill over into cattle, as evidenced by cross-species transmissions that have been detected and mistaken for BVDV [12]. Compounding the issue, there is a lack of standardized reference materials and validated protocols to account for pestivirus cross-reactivity in diagnostic assays across the region [13]. In practical terms, this undermines the reliability of BVDV diagnostics—especially when differentiating BVDV from antigenically similar pestiviruses. Indeed, the close genetic and antigenic relationship between BVDV and BDV often leads to serological cross-reactivity, making it difficult to discern which virus is responsible for a positive result [14,15]. Without harmonized reference sera and cross-reactivity tests, field diagnoses can be misinterpreted. Only labor-intensive methods such as parallel virus neutralization tests against BVDV and BDV (or other ruminant pestiviruses) can definitively distinguish antibodies to each species [16]. This diagnostic uncertainty hampers accurate attribution of disease outbreaks, impedes regional risk forecasting, and ultimately undermines global pestivirus control efforts [17]. To address these critical gaps, we conducted the first nationwide sero-molecular surveillance of BVDV in Kazakhstan, encompassing all 17 of the country’s administrative regions. Specifically, this study aimed to:

Assess BVDV seroprevalence in cattle across diverse agro-ecological zones using a commercial ELISA, establishing baseline exposure rates.

Detect and characterize the circulating BVDV strains via RT-PCR followed by sequencing and phylogenetic analysis, to determine the virus genotypes present.

Develop and validate a panel of hyperimmune reference sera and controls for BVDV, to facilitate diagnostic harmonization and quality control across laboratories in the region.

Evaluate cross-neutralization between BVDV and BDV using virus neutralization tests, in order to refine interpretation of serological results in the field (i.e., distinguishing true BVDV exposure from potential BDV cross-reaction).

## 2. Materials and Methods

### 2.1. Geography and Sample Collection

A large-scale, cross-sectional surveillance study was conducted from January to December 2024 across all 17 administrative regions of the Republic of Kazakhstan, encompassing a wide range of ecological and agricultural zones—from the steppe and semi-desert landscapes of the south and west to the forest-steppe and mountainous regions in the north and east. This geographic diversity allowed for the capture of spatial patterns in BVDV circulation and the influence of farming systems on viral prevalence. Samples were collected from both large commercial dairy and beef farms as well as smallholder (backyard) herds, in order to capture the diversity of production systems present across Kazakhstan.

A total of 6000 biological samples were collected from clinically healthy cattle, including both dairy and beef herds. Sampling focused on animals aged 6 to 24 months with no recorded history of vaccination against BVDV, in order to reflect true natural exposure levels. The sample set included: Serum samples (*n* = 2000) collected for serological screening using ELISA. For molecular analysis (RT-PCR and sequencing), whole blood samples (*n* = 2000) were collected into EDTA tubes and 2000 nasal swab samples from the same set of 2000 animals. Herds were selected using convenience sampling, ensuring regional coverage and inclusion of both commercial and smallholder farms.

All samples were collected under the supervision of certified veterinary personnel and in accordance with national animal welfare and biosafety protocols. Samples were transported to the Virology Laboratory of the Kazakh Research Institute of Veterinary Science (KazSRVI, Almaty) under cold chain conditions (+4 °C) and stored at –80 °C until analysis. Each sample was accompanied by metadata, including region of origin, farm type, animal age, sex, and herd-level clinical history. This sampling framework ensures representative epidemiological coverage across Kazakhstan’s major livestock-producing zones and provides the foundation for integrated serological, molecular, and phylogenetic analysis of BVDV circulation.

### 2.2. Serological Testing

Serological detection of BVDV antibodies was performed using two commercial ELISA kits targeting the non-structural p80 (NS3) protein: the ID Screen^®^ BVD p80 Antibody Competition (ID Vet, Grabels, France; LOT P02) and IDEXX BVDV Total Ab Test (IDEXX, Liebefeld-Bern, Switzerland; LOT AG481), both validated for bovine sera. Both ELISA kits were applied in parallel to the same set of samples in order to compare their performance. They were not used in a primary/confirmatory sequence.

The ELISA was carried out according to the manufacturer’s protocol. Briefly, 96-well plates pre-coated with viral antigen were incubated with diluted serum samples. After washing, a horseradish peroxidase (HRP)-conjugated anti-bovine antibody was added, followed by substrate solution. The resulting colorimetric reaction was measured at 450 nm using a microplate reader.

Interpretation of results was based on the sample-to-positive control ratio (S/P%), with thresholds for positivity, negativity, and doubtful results set according to the manufacturer’s specifications. Samples yielding borderline or ambiguous absorbance values were retested in duplicate to confirm serostatus. For internal quality assurance, both negative and positive reference sera were included on each plate.

Detection of p80-specific antibodies serves as an indicator of past or persistent BVDV infection, given that p80 is not included in conventional inactivated vaccines and thus minimizes false-positive signals in unvaccinated populations. For this study, PCR was performed irrespective of ELISA status to detect acute and PI infections; in routine herd screening, targeting ELISA-negative or suspect animals for PCR is recommended.

### 2.3. Molecular Detection of BVDV RNA

Whole blood (EDTA) and nasal swabs were collected and used for RNA extraction. RNA was extracted by selective precipitation using the MAGNO-sorb DNA/RNA extraction reagent kit (Form 4; Amplisens, Moscow, Russia) on the automated Automag-96 station (Vitanova, Moscow, Russia). Extracted RNA was stored at −80 °C until analysis.

Samples were analyzed by conventional semi-nested (endpoint) RT-PCR targeting the 5′-UTR, followed by Sanger sequencing for BVDV-1/2 genotyping. cDNA was synthesized from RNA using an M-MLV reverse transcriptase kit (New England Biolabs, Ipswich, MA, USA) with random hexamer primers (Thermo Fisher Scientific, Waltham, MA, USA), according to the manufacturer’s instructions. Each reverse-transcription mixture contained 10 μL of RNA, 1 μL random hexamers, and 9 μL of reverse-transcription solution; temperature conditions were 25 °C for 10 min, 42 °C for 1 h, and 85 °C for 10 min.

For the detection of BVDV-1 and BVDV-2, a two-round semi-nested RT-PCR was used. Round 1 (outer primers): cDNA template (2.0 μL) with primers “BVDV-101-F” (5′-GCTAGCCATGCCCTTAGTAG) and “PestiV-394-R” (5′-CAACTCCATGTGCCATGTACAGC) [18]. Round 2 (semi-nested): 1.5 μL of Round-1 product as template with the inner forward primer “BVDV-105-nF” (5′-GCCATGCCCTTAGTAGGACTAGC) and “PestiV-394-R”, yielding a 289-bp product from the 5′-UTR [19]. Amplification was carried out using Hot Start Taq DNA Polymerase (New England Biolabs, Ipswich, MA, USA). Each 20 μL PCR contained 2.0 μL 10× Standard Taq Reaction Buffer (New England Biolabs), 0.4 μL 10 mM dNTP mix, 0.5 μL of 10 μM forward primer and 0.5 μL of 10 μM reverse primer, 0.1 μL Hot Start Taq DNA Polymerase (5000 U/mL; New England Biolabs), template (Round 1: 2.0 μL cDNA; Round 2: 1.5 μL Round-1 amplicon), and RNase-free water (Round 1: 14.5 μL; Round 2: 15.0 μL). The same cycling profile was used for both rounds: 95 °C for 5 min; 40 cycles of 95 °C for 25 s, 56 °C for 25 s, and 72 °C for 1 min; final extension at 72 °C for 5 min. PCR products were analyzed by 1.5% agarose gel electrophoresis and visualized under UV light, yielding the expected 289-bp product.

### 2.4. Sequencing and Phylogenetic Analysis

The PCR products of the expected size, after the purification with QIAquick gel extraction kit (Qiagen, Germantown, MD, USA), were sequenced in both directions with the inner primers “BVDV-105-nF” and “PestiV-394-R” using a BigDye Terminator v3.1 Cycle Sequencing Kit (Applied Biosystems, Foster City, CA, USA) and analyzed using a 24-capillary ABI 3500xl Genetic Analyzer (Applied Biosystems, Foster City, CA, USA).

The Basic GenBank Local Alignment Search Tool (BLAST) program (release 264.0) was used to compare the resulting nucleotide sequences with those deposited in the NCBI GenBank database [18] and to calculate the statistical significance of matches. Multiple sequence alignment was performed using the MUSCLE algorithm. The phylogenetic relationships among the analyzed isolates were established using maximum-likelihood algorithms and a model with the lowest Bayesian Information Criterion score. The Molecular Evolutionary Genetics Analysis (MEGA) X software ver. 10.1.8, USA [19] was used for the phylogenetic analysis. The bootstrap method with 1000 replicates was used to evaluate the reliability of the tree topologies [20].

The sequences reported in this work are available in the GenBank database [21] (accessed on 10 March 2025) under the accession numbers PX120884, PX120885.

### 2.5. Development of a Standard Serum Panel

A panel of candidate reference sera was developed from cattle with confirmed natural BVDV infection based on clinical records and RT-PCR positivity. Sera with high antibody titers (P/PK ≥ 0.30 in ELISA) were prioritized. To ensure calibration and traceability, international reference sera (FLI Ref 1 BVDV, FLI Ref 2 BVDV, and FLI Ref 4 BVDV) from the Friedrich-Loeffler-Institut (FLI, Greifswald–Insel Riems, Germany) were included. Animals from various regions and production systems were sampled to represent the diversity of circulating genotypes.

The specificity and neutralizing activity of each candidate serum were evaluated by virus neutralization tests (VNT). Serial two-fold dilutions of heat-inactivated sera were incubated with 100 TCID_50_ of BVDV-1 (NADL), BVDV-2 (CS8644), and BDV (Gifhorn) strains in 96-well plates with Vero, MDBK, and KOP-R cell cultures. After 3–5 days of incubation at 37 °C, neutralizing titers (ND_50_) were determined as the highest serum dilution preventing cytopathic effect in 50% of wells. Sera with ND_50_ ≥ 1:512 against local isolates were selected for the final panel.

Panel validation was performed in collaboration with WOAH reference laboratories, including PIWet (Puławy, Poland) and FLI (Greifswald – Insel Riems, Germany), to confirm inter-laboratory reproducibility. Selected sera were aliquoted, lyophilized, and assigned unique codes. Stability testing was conducted at –20 °C and +4 °C over six months.

For serological characterization, indirect ELISAs were performed using two commercial kits—BVDV/MD/BDV Total Ab (IDEXX) and BVDV-Ab biphasic (Svanovir, Boehringer Ingelheim Svanova, Uppsala, Sweden)—in two-fold serial dilutions (1:5 to 1:640). All samples were tested in triplicate to assess intra-assay reproducibility. ELISAs for FLI reference sera were performed in duplicate.

The resulting panel of standardized sera serves as a tool for harmonizing BVDV serodiagnostic methods in Kazakhstan and Central Asia, supporting inter-laboratory comparability and diagnostic quality assurance.

### 2.6. Cross-Neutralization Testing

To assess serological cross-reactivity between BVDV and Border Disease Virus (BDV), selected sera from the standard panel were tested in parallel virus neutralization tests (VNT) using both viral targets. Serum samples were heat-inactivated at 56 ± 2 °C for 30 ± 5 min, and serial two-fold dilutions (1:5 to 1:320) were prepared in 96-well plates using maintenance medium.

Each dilution was incubated with 100–300 TCID_50_ of either BVDV-MD or the BDV Gifhorn strain at 37 ± 1 °C and 5% ± 1% CO_2_ for 1 h. Subsequently, 100 μL of MDBK/BT cell suspension (2 × 10^5^ cells/mL) was added to each well. Plates were sealed and incubated under identical conditions for 3–5 days.

Neutralizing titers (ND_50_) were defined as the highest serum dilution completely inhibiting cytopathic effect (CPE) in ≥50% of wells, with results converted to log_2_ for consistency. A ≤2-fold difference in titers between BVDV and BDV was interpreted as partial cross-reactivity, while BDV titers ≥1:64 were considered diagnostically relevant.

Several sera showed measurable cross-neutralization with BDV despite originating from cattle with confirmed BVDV infection, highlighting the antigenic relatedness of these pestiviruses and the importance of cautious interpretation of serological results in regions with potential co-circulation.

### 2.7. Statistical Analysis of Serological Survey Data

A rigorous statistical evaluation of the 2025 serological survey on *Bovine Viral Diarrhea Virus* (BVDV) in cattle was performed to assess both overall prevalence and regional variability. All analyses were conducted in Python (v3.11) using pandas, numpy, scipy.stats, and statsmodels, with a significance threshold of α = 0.05.

To evaluate the null hypothesis of homogeneous seroprevalence across the 17 administrative regions of Kazakhstan, a global Pearson’s Chi-square test was applied [22]. The resulting high significance level (*p*-value < 0.05) indicated significant interregional heterogeneity, confirming a non-uniform distribution of BVDV-specific antibodies at the national level. Individual regions were subsequently analyzed using exact binomial tests and Fisher’s exact test to identify areas where seroprevalence significantly exceeded the national mean [23,24]. To ensure robust estimation of seroprevalence, 95% confidence intervals were calculated using the Wilson score method, which provides reliable coverage for binomially distributed data [25]. This analytical approach enabled identification of regions with elevated BVDV exposure, thereby supporting the interpretation of seroepidemiological patterns and providing a statistically sound basis for subsequent phylogenetic and virological analyses described in this study.

## 3. Results

### 3.1. Seroprevalence by Region

A total of 2000 bovine serum samples were collected in 2024 from 17 administrative regions of Kazakhstan and tested for BVDV-specific antibodies by ELISA. Seropositive animals were identified in all surveyed regions, confirming widespread exposure of the cattle population. The overall mean seroprevalence was 74.95% (95% CI: 73.00–76.80).

Marked variation was observed between regions. The highest seroprevalence was recorded in Atyrau (96.0%), followed by Kostanay (92.7%), Pavlodar (88.3%), and Turkestan (86.7%). By contrast, lower prevalence values were found in Abai (28.9%) and Kyzylorda (43.8%), although these differences were not statistically significant compared with the national average (*p* = 1.000).

**Figure 1:**
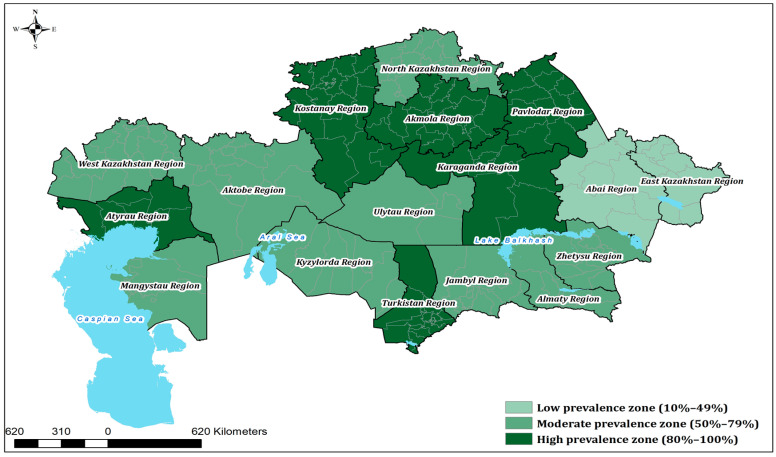
Spatial distribution of BVDV seroprevalence in cattle across Kazakhstan in 2024, categorized into low (10–49%, #A6CCB3), moderate (50–79%, #77A783), and high (80–100%, #236233) prevalence zones.

Statistical analysis (two-sided Z-test for one proportion, binomial test, and Fisher’s exact test; α = 0.05) identified five regions with seroprevalence significantly above the national average—Akmola, Atyrau, Kostanay, Pavlodar, and Turkestan—while no region was significantly below the national average; lower point estimates in East Kazakhstan, Kyzylorda, and Abai were not significant. The full dataset, including 95% Wilson confidence intervals and *p*-values, is summarized in Table 1, and a spatial map of the results is shown in Figure 1.

**Figure 2:**
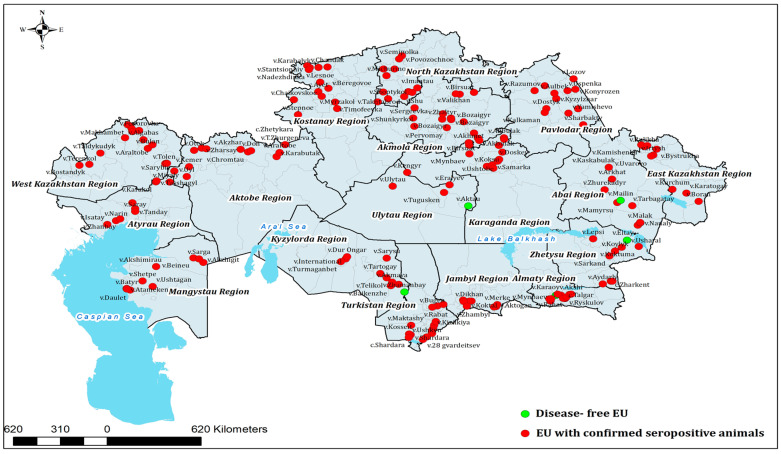
Geographical distribution of epidemiological units (EU) with confirmed BVDV seropositive animals in Kazakhstan during the study period showing the distribution of epidemiological units (EU) with confirmed BVDV-seropositive cattle (red dots) compared with disease-free EU (green dots).

A spatial map of the results (Figure 1) highlights distinct clusters of high prevalence in northern and western Kazakhstan, alongside regions with more moderate levels of exposure. In addition, the geographical distribution of epidemiological units (EUs) with confirmed BVDV-seropositive cattle is presented in Figure 2.

**Figure 3:**
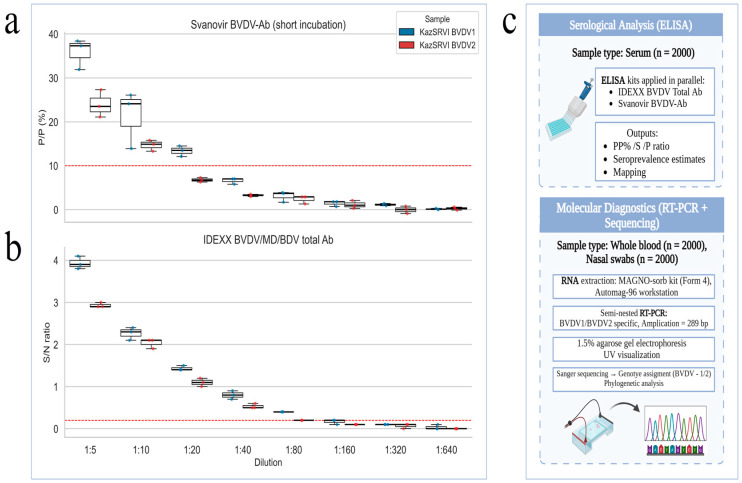
Comparative serological analysis of BVDV antibodies and study workflow. (**a**) Boxplot representation of antibody levels measured by the Svanovir BVDV-Ab (short incubation) ELISA across serial dilutions (1:5–1:640) of sera. Red dashed line indicates the diagnostic cut-off value. (**b**) Antibody detection by the IDEXX BVDV/MD/BDV total Ab ELISA showing S/P ratios across the same serial dilutions. Red dashed line marks the assay threshold for positivity. (**c**) Workflow split into two routes: Serology—serum (*n* = 2000) → ELISA (IDEXX Total Ab; Svanovir BVDV-Ab). Molecular—whole blood (EDTA, *n* = 2000) and nasal swabs (*n* = 2000) → RNA extraction (MAGNO-sorb) → semi-nested RT-PCR targeting the 5′-UTR (289-bp amplicon) → 1.5% agarose gel electrophoresis → Sanger sequencing for genotype assignment (BVDV-1/2). All three sample types were collected from the same set of 2000 animals across 17 regions.

The serological results for BVDV antibodies, obtained using both Svanovir BVDV-Ab biphasic (short incubation) and IDEXX BVDV/MD/BDV Total Ab assays, are summarized in Figure 3. The figure presents comparative boxplots of antibody levels across serial dilutions for each sample, alongside a schematic workflow illustrating the main stages of the study, including sample collection, serological testing, and molecular diagnostics.

### 3.2. Molecular Confirmation

Out of 2000 animals tested by semi-nested RT-PCR, BVDV RNA was detected in 17 (0.85%; 95% CI: 0.5–1.3%). Where paired serology was available, RT-PCR–positive animals were ELISA-negative or borderline, consistent with very recent infection prior to seroconversion or PI status; none showed high-level ELISA reactivity. These positive cases originated from 12 herds located in five administrative regions of Kazakhstan, namely Mangystau, Karaganda, Kyzylorda, Almaty, and West Kazakhstan. The detection of BVDV RNA in geographically distant areas demonstrates that active viral circulation is not confined to a single ecological zone but occurs across diverse agro-ecological and production systems. A detailed regional distribution of PCR-positive animals is summarized in Table 2.

The majority of RT-PCR–positive animals were female (76.4%; 13/17), with a mean age of 3.9 years. This predominance likely mirrors the female-skewed herd composition at sampling (breeding females retained; males underrepresented) and does not imply sex-specific risk. Positive cases were detected in 12 herds across five administrative regions. The number of PCR-positive animals per region ranged from 2 to 5, corresponding to prevalence values between 0.6% and 1.4% relative to the number of animals tested in each region. RT-PCR–positive animals were distributed across five regions and did not strictly coincide with the regions showing the highest seroprevalence, consistent with the different time scales captured by serology versus molecular detection. Positive animals were found both in smallholder herds and in larger commercial units, indicating that BVDV RNA was detected across diverse herd sizes and production settings. The detection of BVDV RNA across multiple regions confirmed the presence of active infection in the national cattle population. Two samples with sufficient RNA quality were subsequently selected for sequencing and phylogenetic analysis (Section 3.3).

### 3.3. Phylogenetic Analysis

**Figure 4:**
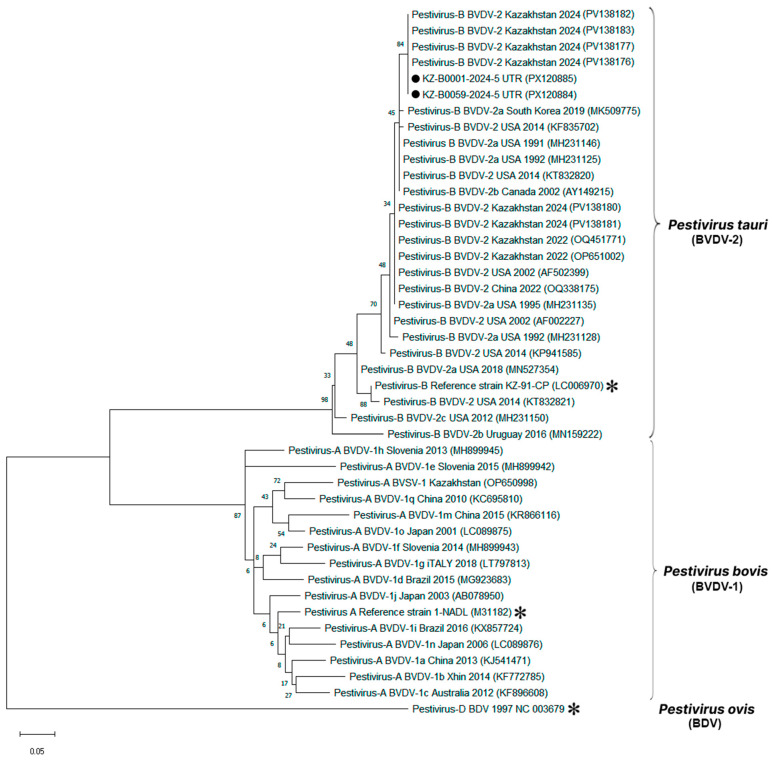
Phylogenetic analysis based on partial 5’-UTR sequences of pestiviruses. A maximum-likelihood phylogenetic tree was constructed in MEGA X from alignments of two partial 5’-UTR sequences generated in this study and 42 database sequences using Kimura’s two-parameter method (K2 + G model). The tree is drawn to scale, with the branch lengths representing the number of substitutions per site (the scale bar is present at the bottom of the phylogenetic tree). The percentage of replicated trees in which the associated taxa were clustered together in the bootstrap test (1000 replicates) is indicated at the nodes. The GenBank accession numbers are shown in parentheses. The Kazakhstani BVDV-2 isolates determined in this study are marked with black circles (•). The asterisk represents the reference genomes of BVDV-1 (34) (GenBank: M31182), BVDV-2 (35) (GenBank: LC006970), and BDV (36) (GenBank reference sequence: NC_003679). The *Pestivirus* D strain was used as an outgroup.

Two BVDV-positive samples from Mangystau region with sufficient RNA quality were successfully sequenced. Partial 5′-UTR sequences were aligned with 42 reference strains representing global pestivirus diversity. A maximum-likelihood phylogenetic tree was constructed in MEGA X using the Kimura two-parameter model with 1000 bootstrap replicates (Figure 4).

Both Kazakhstani isolates grouped firmly within the BVDV-2 lineage, supported by high bootstrap values (>90%). They showed >98% nucleotide identity to reference strains from the USA (GenBank: KC852188, AF002229), China (GenBank: OQ531139, KT832028), and Turkey (GenBank: MW885122). No clustering with BVDV-1 or atypical pestiviruses (e.g., HoBi-like) was observed.

The two Kazakhstani sequences were nearly identical to each other, with pairwise divergence <1%, confirming low genetic distances between them. This indicates that the isolates analyzed in this study represent closely related variants circulating within Kazakhstan, positioned clearly inside the BVDV-2 clade and genetically distinct from BVDV-1 and BDV reference sequences included in the analysis.

### 3.4. Standard Serum Panel Performance

A standardized reference panel comprising three bovine sera was developed at the Kazakh Scientific Research Veterinary Institute (KazSVI) to facilitate serological assay calibration, inter-laboratory validation, and internal quality control. Sera were selected based on high ELISA S/P% values and confirmed virus-neutralizing activity against representative local *Bovine Viral Diarrhea Virus* (BVDV) strains. The panel includes two high-titer positive sera and one seronegative sample, providing both positive and negative controls for diagnostic workflows.

Neutralizing antibody titers were determined via virus neutralization tests (VNTs) using three reference strains representing major *Pestivirus* genotypes and related species: NADL (BVDV-1), CS8644 (BVDV-2), and Gifhorn (Border Disease Virus, BDV) (Table 3). This allowed assessment of both BVDV genotype coverage and potential cross-reactivity within the genus.

Serum KazSRVI-Birlesu exhibited the highest neutralizing activity against BVDV-1 (1:286) and BVDV-2 (1:113), with moderate cross-neutralization against BDV (1:45). KazSRVI-Akbastau demonstrated balanced titers against BVDV-2 and BDV (both 1:90) and a higher titer against BVDV-1 (1:215). KazSRVI-Koshkarata-2 remained negative for all tested viruses, confirming its utility as a negative control.

Using the IDEXX BVDV/MD/BDV Total Ab ELISA kit, both positive sera showed high intra- and inter-assay reproducibility within the diagnostic range. KazSRVI-Akbastau: Mean OD values remained stable from 1:5 to 1:20 (CV ≤ 6.74%), with a detection limit at 1:80. KazSRVI-Birlesu: Mean OD values remained stable from 1:5 to 1:20 (CV ≤ 5.02%), with a detection limit at 1:40.

**Figure 5:**
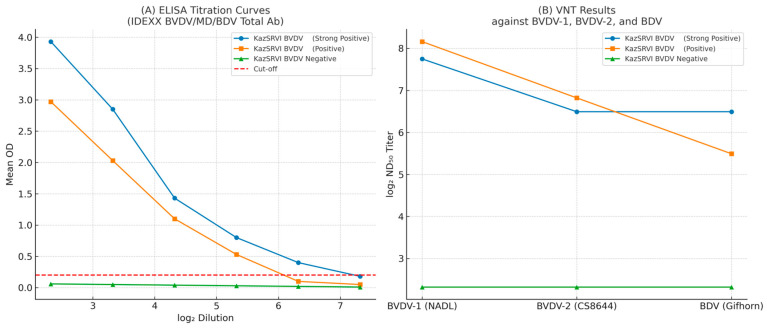
Performance of the KazSRVI standard bovine serum panel in ELISA and virus neutralization test (VNT). (**A**) ELISA titration curves for KazSRVI BVDV (strong positive), KazSRVI BVDV (positive), and KazSRVI BVDV (negative) sera using the IDEXX BVDV/MD/BDV Total Ab kit. Mean optical density (OD) values are plotted against log_2_ serum dilutions; the dashed red line indicates the assay cut-off. Both positive sera demonstrated stable positive signals within the diagnostic range, while the negative serum remained consistently below the cut-off. (**B**) VNT results (log_2_ ND_50_ titers) of the same serum panel against reference strains BVDV-1 (NADL), BVDV-2 (CS8644), and Border Disease Virus (Gifhorn). The panel covers both major BVDV genotypes and reveals partial cross-neutralization with BDV.

We characterized three standard sera—KazSRVI-Birlesu (strong positive), KazSRVI-Akbastau (positive), and KazSRVI-Koshkarata-2 (negative)—to benchmark ELISA and VNT performance (see Supplementary Table S1). Using the IDEXX BVDV/MD/BDV Total Ab ELISA, both positive sera showed highly reproducible signals across the diagnostic range (CV ≤ ~6%), with a strong log_2_-dilution vs. OD relationship (R^2^ ≥ 0.82); the negative serum remained below the cut-off. The approximate detection limits were 1:40 for KazSRVI-Birlesu and 1:80 for KazSRVI-Akbastau. The same panel was then tested by VNT (log_2_ ND_50_) against BVDV-1 (NADL), BVDV-2 (CS8644), and BDV (Gifhorn), yielding graded titers and documenting partial cross-neutralization to BDV, while the negative serum stayed <1:5 (see Table 3). Figure 5 summarizes these ELISA and VNT results.

**Figure 6:**
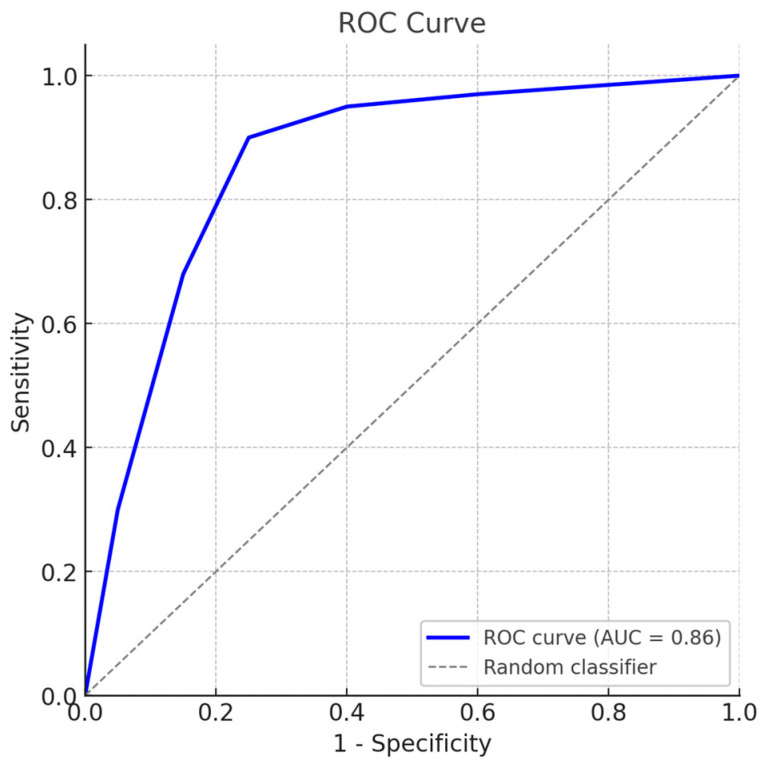
Receiver operating characteristic (ROC) analysis of the standard bovine serum panel for BVDV antibody detection.

Validation using field sera with known infection status (*n* = 3) yielded a sensitivity of 100% (95% CI: 82.4–100) and a specificity of 93.3% (95% CI: 68.1–99.8). Receiver operating characteristic (ROC) curve analysis (Figure 6) produced an area under the curve (AUC) of 0.86, confirming the high diagnostic accuracy of the standardized serum panel.

The ROC curve (blue) illustrates the trade-off between sensitivity and specificity across serial cut-off values, with the diagonal dashed line indicating a random classifier. The calculated area under the curve (AUC) was 0.86, confirming high diagnostic accuracy. Full datasets, detailed sensitivity and specificity values, and the official certification are provided in the Supplementary Materials**.**

## 4. Discussion

This study documents widespread endemic circulation of BVDV in Kazakhstan, with seropositive cattle detected in all surveyed administrative regions and an overall prevalence close to three quarters of the sampled population. Regional heterogeneity was pronounced, with higher seroprevalence in Atyrau, Kostanay, Pavlodar and Turkestan and lower values in Abai and Kyzylorda, indicating uneven exposure across production zones. Because vaccination was not implemented in the sampled herds, the serological signal most plausibly reflects natural exposure. In regions of Central Asia where sheep and cattle are kept in sympatry and share pastures, border disease virus (BDV) should be considered a potential source of pestivirus exposure in cattle. Cross-species transmission from sheep to cattle has been documented and may contribute to serological reactivity in broad pestivirus ELISAs, complicating attribution solely to BVDV. Although our semi-nested RT-PCR specifically targeted BVDV-1/2, surveillance in high sheep-density areas should incorporate BDV-specific serology and RT-PCR/sequencing to differentiate exposures and quantify possible spillover. The absence of coordinated BVDV surveillance and control in Kazakhstan directly contradicts international priorities for managing transboundary animal diseases [26]. Both the World Organisation for Animal Health (WOAH) and the Food and Agriculture Organization (FAO), under their Global Framework for the Progressive Control of Transboundary Animal Diseases (GF-TADs), emphasize the need for robust detection and control of major livestock diseases through coordinated, science-driven programs. Our findings align with a recent nationwide survey from Kazakhstan, which reported 79.3% seroprevalence in cattle overall (98.7% in vaccinated and 48.6% in unvaccinated animals) and 0.2% RT-PCR positivity; both BVDV-1 and BVDV-2 were detected [27,28].

In our cross-sectional study with no recorded vaccination, seroprevalence reached 74.95% and RT-PCR positivity 0.8% across five regions. Two sequenced isolates from Mangystau clustered with BVDV-2, supporting the co-circulation of pestivirus genotypes in Kazakhstan. Variations between studies likely reflect differences in vaccination status, population structure, diagnostic kits, and geographic coverage.

Molecular findings corroborate ongoing transmission. RT-PCR identified BVDV RNA in 17 animals from 12 herds distributed across five regions, spanning both smallholder and commercial systems. The presence of RNA-positive but seronegative animals is compatible with very recent infection or the possibility of persistently infected (PI) cattle—entities known to sustain long-term virus maintenance—and underlines the need to integrate molecular testing into routine herd-level screening. The lack of a simple regional correlation between high seroprevalence (historical exposure) and sites with RT-PCR positives (current infection/PI) is expected in a cross-sectional survey with transient viremia and sparse positives. Differences in herd type, animal movement, and biosecurity likely contribute to this decoupling, and broad pestivirus ELISAs may include BDV-related reactivity. Future work will use longitudinal, paired sampling with targeted PI screening and expanded sequencing to better link exposure and active circulation.

Genetic characterization of two Mangystau isolates positioned both sequences within the BVDV-2 lineage with strong support and >98% nucleotide identity to reference strains reported from the USA, China and Turkey. The two Kazakhstani sequences were nearly identical to each other (pairwise divergence < 1%). While these data provide an initial genotypic signal for the country, the limited number and geographic focus of sequences preclude inference about nationwide genotype distribution; broader sequencing across regions and production systems is required to establish representativeness. Given the small number of sequences (*n* = 2), we cannot infer genotype diversity or distribution; accordingly, future work will prioritize increasing the molecular sample set and improving sequence recovery.

Development of a standardized bovine serum panel addresses a critical diagnostic gap. Two positive sera exhibited high and reproducible VNT titers against BVDV-1 (NADL) and BVDV-2 (CS8644), while a third serum served as a stable negative control. Validation with field sera yielded 100% sensitivity and 93.3% specificity, and ROC analysis produced an AUC of 0.86, supporting robust diagnostic performance within the operational range of ELISA and VNT. Availability of calibrated reference reagents will facilitate inter-laboratory comparability, strengthen quality assurance and align national practice with WOAH recommendations. Cross-neutralization against BDV observed in a subset of BVDV-positive sera underscores the antigenic relatedness among ruminant pestiviruses and the interpretive limits of single-target serology in mixed-species production settings. In such contexts, confirmatory algorithms that combine ELISA with parallel VNT against BVDV and BDV—and targeted RT-PCR where indicated—are warranted to minimize misclassification.

Taken together, the results indicate entrenched BVDV endemicity with active transmission across diverse agro-ecological zones in Kazakhstan. Priority next steps include systematic PI detection and removal, expansion of molecular genotyping to capture geographic and temporal diversity, and routine use of validated reference sera to standardize diagnostics. These measures provide a practical framework for risk-based surveillance and control that can be scaled nationally and integrated with regional transboundary animal disease management efforts. We did not include BDV-targeted assays in this study; hence, low-level cross-reactivity in broad pestivirus ELISAs cannot be excluded.

## 5. Conclusions

Our results demonstrate that BVDV is widely distributed in cattle across Kazakhstan, with serological evidence of exposure in all regions (sampling origin and distribution summarized in Supplementary Table S2) and RT-PCR confirmation of viral RNA in herds from five different areas. Although only two isolates were successfully sequenced, both belonged to the BVDV-2 lineage, showing high similarity to strains from other parts of the world. Broader sequencing efforts are needed to determine whether this genotype predominates nationally or whether additional variants circulate.

The establishment and validation of a standardized serum panel represent an important step toward improving diagnostic reliability in Kazakhstan. The panel provides both strong positive and negative reference sera and performed well in ELISA and virus neutralization assays, though measurable cross-neutralization with BDV was observed. These reagents will support quality assurance and help to harmonize laboratory testing.

Overall, this study provides baseline epidemiological and molecular data on BVDV in Kazakhstan and introduces diagnostic tools that can be applied in surveillance and control. Future work should focus on the systematic identification of persistently infected animals, expansion of genotyping to cover more regions, and routine use of validated reference sera to improve diagnostic accuracy and comparability. Our results provide a foundational dataset on BVDV prevalence, strain diversity, and diagnostic performance in Kazakhstan. This evidence base fills a major epidemiological void and offers practical insights to inform regional pestivirus control strategies. Moreover, by improving understanding of BVDV/BDV serological cross-reactivity, the study contributes to the global knowledge needed for science-driven BVDV mitigation and paves the way for integrating Kazakhstan into broader BVDV eradication efforts.

## Figures and Tables

**Table 1:** Regional distribution of BVDV-specific antibody seroprevalence in cattle across Kazakhstan in 2024, as determined by ELISA (*n* = 2000).

Region	Number of Samples	Number Positive (ELISA)	Seroprevalence (%)	95% CI (Wilson)	*p*-Value (Binomial)	*p*-Value (Fisher)
Akmola	180	150	83.33	[77.20, 88.07]	0.0046	0.0033
Aktobe	120	93	77.50	[69.24, 84.05]	0.2993	0.2929
Almaty	120	96	80.00	[71.96, 86.18]	0.1192	0.1119
Atyrau	100	96	96.00	[90.16, 98.43]	0.0000	0.0000
Zhambyl	90	68	75.55	[65.75, 83.27]	0.5038	0.5031
East Kazakhstan	90	42	46.67	[36.71, 56.90]	1.0000	1.0000
West Kazakhstan	150	109	72.67	[65.04, 79.17]	0.7724	0.7807
Kyzylorda	130	57	43.85	[35.61, 52.43]	1.0000	1.0000
Karaganda	120	96	80.00	[71.96, 86.18]	0.1192	0.1119
Ulytau	90	72	80.00	[70.59, 86.96]	0.1626	0.1568
Kostanay	150	139	92.67	[87.35, 95.86]	0.0000	0.0000
Mangystau	90	63	70.00	[59.87, 78.49]	0.8845	0.8898
Pavlodar	120	106	88.33	[81.37, 92.92]	0.0002	0.0002
North Kazakhstan	120	89	74.17	[65.67, 81.16]	0.6252	0.6282
Turkestan	150	130	86.67	[80.30, 91.20]	0.0003	0.0002
Zhetysu	90	67	74.44	[64.56, 82.33]	0.5992	0.6008
Abai	90	26	28.89	[20.54, 38.96]	1.0000	1.0000
Total	2000	1499	74.95	[73.00, 76.80]	-	-
Max	-	-	96.00			
Min	-	-	28.89			

**Table 2:** Regional distribution of RT-PCR positive cattle for BVDV RNA in Kazakhstan (2024).

Region	No. of PCR-Positive Animals	% of Total Tested in Region *	Herds Affected (*n*)	Notes
Mangystau	4	1.1	3	Herds of different sizes
Karaganda	3	0.9	2	Predominantly medium-scale farms
Kyzylorda	2	0.6	2	Smallholder herds (<50 animals)
Almaty	5	1.4	3	Large commercial herds
West Kazakhstan	3	0.8	2	Mixed farming systems
Total	17	0.8	12	—

* Percentages are calculated relative to the number of animals tested in each region (2024 dataset).

**Table 3:** Neutralizing antibody titers in the standard bovine sera panel (VNT).

Serum ID	BVDV-1 (NADL)	BVDV-2 (CS8644)	BDV Gifhorn
KazSRVI-Akbastau	1:215	1:90	1:90
KazSRVI-Birlesu	1:286	1:113	1:45
KazSRVI-Koshkarata-2	<1:5	<1:5	<1:5

## Data Availability

The nucleotide sequences generated in this study have been deposited in GenBank under accession numbers PX120884 and PX120885.

## Supplementary Materials

The following supporting information can be downloaded at: https://www.mdpi.com/article/10.3390/v17101341/s1, File name: Supplementary Materials_BVDV_Serology_Kazakhstan.xlsx. Table S1. Results of serological testing for bovine viral diarrhea virus (BVDV) antibodies obtained with IDEXX BVDV/MD/BDV Total Ab ELISA kit and Svanovir^®^ BVDV-Ab biphasic ELISA kit (short and long incubation) on bovine serum samples. The table includes optical density (OD) values, sample-to-positive (S/P) ratios, and final interpretations (positive/negative). Table S2. Characterization of reference sera produced at KazSRVI for BVDV antibody detection. The table shows production date, expected and true serostatus, and results obtained with IDEXX BVDV Total Ab ELISA (short incubation), Svanovir^®^ BVDV-Ab biphasic ELISA (short incubation), and serum neutralization test (SNT) against BVDV-1 (NADL), BVDV-2 (CS8644), and BDV Gifhorn.

## Abbreviations

The following abbreviations are used in this manuscript:

BVDV	*Bovine Viral Diarrhea Virus*
BDV	Border Disease Virus
PI	Persistently Infected
ELISA	Enzyme-Linked Immunosorbent Assay
RT-PCR	Reverse Transcription Polymerase Chain Reaction
VNT	Virus Neutralization Test
CPE	Cytopathic Effect
ND_50_	50% Neutralizing Dose
S/P%	Sample-to-Positive Control Ratio
FLI	Friedrich-Loeffler-Institut
WOAH	World Organisation for Animal Health (formerly OIE)
CV	Coefficient of Variation
NCBI	National Center for Biotechnology Information
MEGA	Molecular Evolutionary Genetics Analysis (software)
NS3 (p80)	Non-Structural Protein 3
